# Influence of Thermal Boundary Conditions and Number of Channels on the Performance of Heat Sinks with Rectangular Minichannels

**DOI:** 10.3390/mi13081236

**Published:** 2022-07-31

**Authors:** Pamela Vocale

**Affiliations:** Department of Engineering and Architecture, University of Parma, Parco Area delle Scienze 181/A, 43124 Parma, Italy; pamela.vocale@unipr.it

**Keywords:** convective heat transfer, rectangular minichannels, heat transfer enhancement, H1 boundary conditions, H2 boundary conditions, microfluidics

## Abstract

This paper aims to contribute to the analysis of a heat sink designed for the active cooling of small flat surfaces. The heat transfer device investigated here consists of a flat square substrate and a cover, separated by parallel channels with a rectangular cross-section. The cold air flowing in the channels is sucked from the environment, and the bottom of the substrate adheres closely to the hot surface of the device to be cooled. The thermal problem is tackled by considering two different conditions: the first one assuming one long side of the channel is heated and the three other sides are adiabatic (version 1L) and the second one assuming high conductivity of the walls (version 4), in both the H1 and H2 boundary conditions. Moreover, to investigate the effect of the number of channels on the performance of the heat sink, the number of channels is changed between 1 and 20. The results, presented in terms of the *f Re* product, Nusselt number, maximum surface temperature, and thermal resistance, reveal that both the thermal boundary conditions and the number of channels significantly affect the performance of the investigated heat transfer device.

## 1. Introduction

Miniaturized heat sinks have resulted in consistent enhancements to the cooling of many instrume15–20nts and devices. The main advantage offered by heat sinks is the enhanced heat transfer coefficient, which allows the cooled surface to maintain a low temperature even when subjected to a relevant heat flux.

In recent decades, heat sinks with mini and microchannels have been investigated in several works, as highlighted by Elghool et al. [1] and by Dixit and Ghosh [2] in their recent reviews. Due to the importance of the geometric properties, different cross-sections have been investigated, such as rectangular, trapezoidal, or circular cross sections [3,4,5,6,7,8,9]. Particularly, rectangular mini and microchannels are common for easy fabrication; they can be built in silicon wafers by chemical etching or in a solid substrate by a laser-processing system. Rectangular ducts are also employed because they provide a large surface-area-to-volume ratio and enhance the heat transfer coefficients compared with other geometries [10]. 

More recent works were focused on the heat transfer enhancement of mini and microchannel heat sinks. Specifically, Chen et al. [11] proposed a new microchannel heat sink characterized by a cross-rib. Their numerical outcomes revealed that both the thermal performance and the pressure drop increased due to the presence of the cross-rib.

The heat transfer enhancement due to the presence of ribs was also investigated by Tanda and Satta [12] who considered a rectangular channel with longitudinal ribs.

The influence of the aspect ratio and roughness on the performance of sinusoidal rectangular micro- and mini-channel heat sinks was numerically investigated by Ansari and Zhou [13]. They found that both the aspect ratio and the roughness significantly affected the performance of the heat sinks; that is, they concluded that the convective heat transfer could be enhanced by adopting roughness structures and mini and micro-channels with small aspect ratios.

Khonsue [14] carried out an experimental investigation to study the effects of rectangle pin fins, cylindrical pin fins, and spiral pin fins on convective heat transfer in mini and micro heat sinks. His findings highlighted that the heat transfer enhancement due to the presence of fins was significant. 

The influence of fins on the performance of heat sinks was also investigated by Bezaatpour and Goharkhah [15], who proposed a new heat sink characterized by porous fins and magnetite ferrofluid to optimize both the pumping power and the convective heat transfer coefficient.

By adopting a multi-objective genetic algorithm and computational fluid dynamics tools, Ge et al. [16] investigated the length and distribution of fins to optimize the performance of microchannel heat sinks in terms of pressure drop and thermal resistance.

Yang et al. [17] studied the effects of the number of channels and aspect ratio on mini and micro heat sinks characterized by rectangular channels. By considering water and liquid metal as working fluids, they found that the thermal resistance of the heat sink decreased with decreasing aspect ratio, and the pumping power increased as the aspect ratio decreased.

The heat transfer enhancement due to the use of micro-encapsulated phase change materials and nanofluids in mini-channel heat sinks was experimentally investigated by Ho et al. [18]. Their findings revealed that the coefficient of performance, defined as the ratio of the real heat flux to the pumping power, decreased with an increase in the mass fraction of nanoparticles.

The enhancement due to the presence of nanofluids in minichannel heat sinks was also investigated by Saadon et al. [19] who considered five different nanofluids. They found that the silver-water nanofluid guaranteed the lowest value of thermal resistance. Moreover, they investigated the effect of wall corrugation by comparing the performance of wavy channels with conventional channels. The comparison showed an increase in the Nusselt number up to about 50%.

Chen et al. [20] carried out a numerical analysis to study the effects of the triangular prism orientation, height, and distribution on the performance of heat sinks with square minichannels. Their numerical outcomes highlighted that the highest thermal performance could be obtained with a backward triangular prism, although this configuration was characterized by the largest friction coefficient ratio.

Multistage mini-channel heat sinks were numerically investigated by Kim et al. [21] by considering water as a working fluid, they found that the triple stage guaranteed the best thermal performance.

From the previous literature review, it can be deduced that the number of channels significantly affects the performance of the heat sink. It can be also noticed that there are only a few works in which air is considered a working fluid, as highlighted in [13], although air is the most widely used coolant for thermal management of electronic components [22].

Moreover, it must be highlighted that the influence of heat sink materials was investigated by a few researchers. This was due to the fact that heat sinks are usually fabricated in copper, aluminum, or silicon. Nevertheless, because of the high density and electric conductivity of conventional materials, alternative materials, such as high thermal conductivity polymers or ceramics, are considered [22,23].

The effects of heat sink materials can be investigated by imposing different thermal boundary conditions. Several thermal boundary conditions can be considered, as well-known in the literature [24]. Particularly, for channels featuring non-circular cross-sections (e.g. rectangular, elliptical, triangular), which are heated with electric resistance, it is possible to consider four cases, referred to as H1, H2, H3, and H4 [24]. 

Above all, in the present study, only the H1 and H2 boundary conditions were considered. In the H1 boundary condition, the wall heat transfer rate is constant in the axial direction, while the wall temperature is constant in the peripheral direction [24]. In the H2 boundary condition, the wall heat flux is constant in both the axial and peripheral directions [25]. 

From a practical viewpoint, for highly conductive materials (e.g., copper, aluminum), the H1 boundary condition may apply. On the contrary, for materials with low thermal conductivity, such as glass-ceramic or Teflon, the H2 boundary condition may be applied if the wall thickness is uniform.

Moreover, assuming specific conditions for every side of the rectangular channel, eight versions of the thermal boundary conditions can be considered for both H1 and H2 problems [24]. For the thermal problem tackled in the present study, only two versions are worthy of investigation: the 4 version (i.e., all sides of the rectangular channel are heated), 1L version (i.e., the long side of the rectangular channel is the only heated side, with the remaining three sides being adiabatic). These two versions can be obtained if the lateral walls of the channels in the heat sink are metallic (4 version), or if the lateral walls of the channels are materials with low thermal conductivity (1L version).

**Figure 1:**
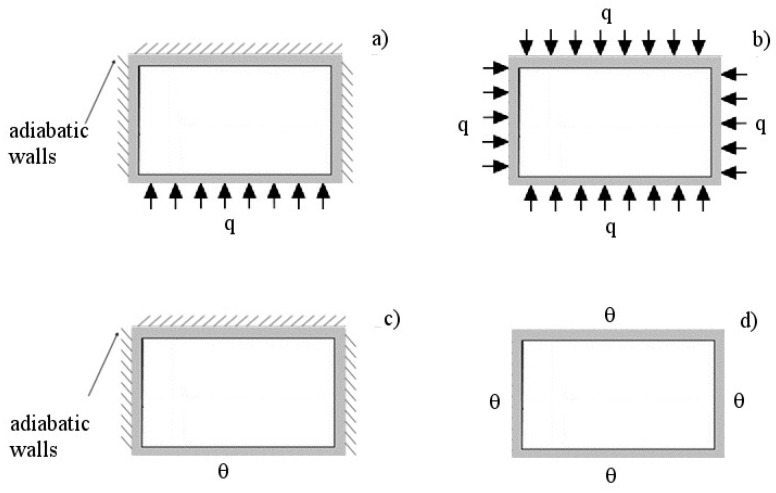
Sketch of the thermal boundary conditions considered in the present study; (**a**) H2,1L; (**b**) H2,4; (**c**) H1,1L; (**d**) H1,4.

Therefore, this work is aimed at investigating the effects of the number of channels and thermal boundary conditions on the performance of heat sinks with rectangular mini/microchannels, by considering air as a working fluid. Particularly, four different conditions were analyzed, namely, H1,4; H1,1L; H2,4; and H2,1L, as shown in Figure 1. For the purpose of the present study, a simplified model of the heat sink is developed and validated against experimental and numerical data available in the literature.

## 2. Mathematical Model

**Figure 2:**
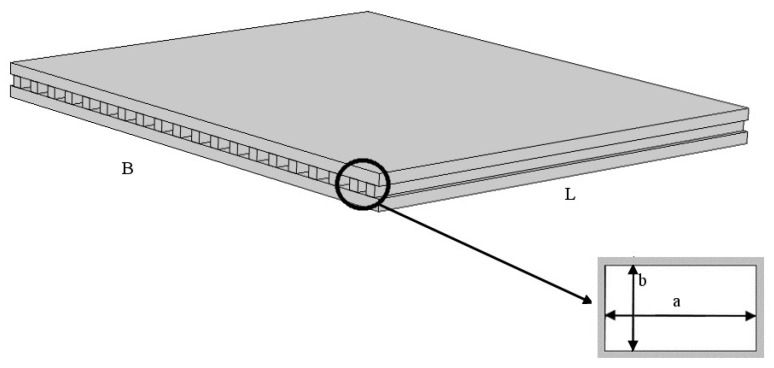
Sketch of the heat sink under investigation.

In the heat sink considered in the present study, a pressure-driven fluid flowed through the minichannels with a rectangular cross section, as shown in Figure 2. The cold air flowing through the channels was sucked from the environment.

The analysis was carried out by considering a single minichannel because symmetry allows to easily extend the results to the entire heat sink [17,26]. The investigated channel was characterized by a rectangular cross-section with longer and shorter sides *a* and *b*, respectively, and length *L*. Because the study was carried out by considering the variable longer side, the aspect ratio *β* = *b*/*a* was also a variable (i.e., *β* ≤ 1).

A Cartesian coordinate system *ξ*, *ψ*, *ζ* was introduced, with its origin in the left bottom corner of the rectangular cross section; *ζ* was horizontal and perpendicular to the channel cross section.

The following hypotheses were assumed:

Newtonian fluid with constant fluid physical properties;
A laminar flow;
Rigid and non-porous walls;
Irrelevant rarefaction, compressibility, roughness, and electrostatic effects; and
Negligible viscous heating and axial conduction.

According to these assumptions, the Navier-Stokes and the energy balance equations were as follows:

(1) $$\mu \left(\frac{\partial^{2}v\left(\xi ,\psi \right)}{\partial \xi^{2}}+\frac{\partial^{2}v\left(\xi ,\psi \right)}{\partial \psi^{2}}\right)=\frac{\partial p}{\partial \zeta }-\rho g_{z}$$

(2) $$\frac{\partial^{2}\theta \left(\xi ,\psi \right)}{\partial \xi^{2}}+\frac{\partial^{2}\theta \left(\xi ,\psi \right)}{\partial \psi^{2}}=\frac{v\left(\xi ,\psi \right)}{\alpha }\frac{\partial \theta }{\partial \zeta }$$

where *v* was the fluid velocity profile, *μ* the fluid dynamic viscosity, $\frac{\partial p}{\partial \zeta }$ the pressure gradient, *θ* the fluid temperature, and *α* the fluid thermal diffusivity.

To obtain a more general solution, the following dimensionless coordinates were introduced:

(3) $$x=\frac{\xi }{a}\;\;\;\;\;\;y=\frac{\psi }{a}\;\;\;\;\;z=\frac{\zeta }{a}$$

as where the following dimensionless functions:

(4) $$V\left(x,y\right)=\frac{v}{W}$$

(5) $$T\left(x,y\right)=\{\begin{array}{c} \frac{\lambda \left(\theta -\theta_{in}\right)}{q^{\prime }}\;\left(H1\;\mathrm{boundary}\;\mathrm{condition}\right)\\ \frac{\lambda \left(\theta -\theta_{in}\right)}{qD_{h}}\;\left(H2\;\mathrm{boundary}\;\mathrm{condition}\right) \end{array}$$

where *W* was the mean velocity of the fluid flow in the channels; *ρ* and *λ* the fluid density and thermal conductivity, respectively; *θ_in_* the inlet fluid temperature; and *q* and *q*’ the linear constant rate and the heat flux, respectively. *D_h_* indicated the hydraulic diameter, which was defined as:

(6) $$D_{h}=\frac{4A}{P}=\frac{2ab}{a+b}\;\;\;\;\;\;\;\;\;\;\;$$

where *A* was the cross-sectional area and *P* the perimeter. 

The Navier–Stokes equation in non-dimensional form was as follows:

(7) $$\frac{\partial^{2}V\left(x,y\right)}{\partial x^{2}}+\frac{\partial^{2}V\left(x,y\right)}{\partial y^{2}}+C=0$$

where *C* was defined as follows:

(8) $$C=\frac{a^{2}}{\mu W}\left(\frac{\partial p}{\partial \zeta }-\varrho g_{\varsigma }\right)$$

with *g_ζ_* being the component of gravity acceleration.

Equation (7) was analytically solved by considering only one rectangular channel resorting to the finite Fourier transform by considering the no-slip boundary condition at the walls [27].

The energy balance equations for the H1 and H2 boundary conditions were as follows:

(9) $$\frac{\partial^{2}T\left(x,y\right)}{\partial^{2}x}+\frac{\partial^{2}T\left(x,y\right)}{\partial y^{2}}=\{\begin{array}{c} \frac{1}{\beta }V\left(x,y\right)\;\;\;\;\left(H1\;\mathrm{boundary}\;\mathrm{condition}\right)\\ \frac{1+\beta }{2\beta^{2}}\frac{P_{h}}{2a}V\left(x,y\right)\;\left(H2\;\mathrm{boundary}\;\mathrm{condition}\right) \end{array}$$

where *P_h_* was the heated perimeter (i.e., *P_h_* =2(*a*+*b*) in conditions 4, while *P_h_*=*a* in the condition 1L).

Equation (9) were analytically solved in [28,29] by considering the 1L version (i.e., only the long side of the rectangular channel on the substrate was heated while the other long side and two short sides were adiabatic) and the 4 version (i.e., all sides of the rectangular channel were heated).

In particular, the boundary conditions for the 1L versions were as follows:

(10) $$\{\begin{array}{c} {\frac{\partial T}{\partial x}|}_{x=0}=0,\;\;\;{\frac{\partial T}{\partial x}|}_{x=1},\;\;\;T\left(x,0\right)=0,\;\;\;{\frac{\partial T}{\partial y}|}_{y=\beta }=0\;\;\;\;\;\left(H1\;\mathrm{boundary}\;\mathrm{condition}\right)\;\\ {\frac{\partial T}{\partial x}|}_{x=0},\;\;\;{\frac{\partial T}{\partial x}|}_{x=1}=0,\;\;\;{\frac{\partial T}{\partial y}|}_{y=0}=-\frac{1+\beta }{2\beta },\;\;\;{\frac{\partial T}{\partial y}|}_{y=\beta }=0\;\left(H2\;\mathrm{boundary}\;\mathrm{condition}\right) \end{array}$$

The boundary conditions for the 4 versions were as follows:

(11) $$\{\begin{array}{c} T\left(0,y\right)=0,\;\;T\left(1,y\right)=0,\;\;T\left(x,0\right)=0,\;\;T\left(x,\beta \right)=0\;\;\;\;\;\;\;\;\;\;\;\;\;\;\;\;\;\;\left(H1\;\mathrm{boundary}\;\mathrm{condition}\right)\\ {\frac{\partial T}{\partial x}|}_{x=0}=-\frac{1+\beta }{2\beta },\;\;\;{\frac{\partial T}{\partial x}|}_{x=1}=\frac{1+\beta }{2\beta },\;\;\;{\frac{\partial T}{\partial y}|}_{y=0}=-\frac{1+\beta }{2\beta },\;\;\;{\frac{\partial T}{\partial y}|}_{y=\beta }=\frac{1+\beta }{2\beta }\;\;\left(H2\;\mathrm{boundary}\;\mathrm{condition}\right) \end{array}$$

The solutions available in the literature [27,28,29] were adopted to evaluate the performance of the heat sinks analyzed in the present study. By fixing the height of the channels (i.e., the shorter side of the channels *b*), changing the number of channels implies changing the aspect ratio of the channels. Therefore, the solution presented in [27,28,29] can be adopted for the present study by considering the assumptions made to obtain these solutions. 

In particular, the thermophysical properties of the working fluid can be considered independent of temperature if the temperature increase of the air in the heat sinks is small. To check the applicability of this assumption, the increase in the bulk temperature of the air must be evaluated, as illustrated later. To check the applicability of the assumption of a fully developed flow, the hydrodynamic and thermal entry lengths must be evaluated. In the literature, few works focused on developing flows in rectangular channels [24,30,31,32,33].

Several different combinations of the main parameters that affect fluid behavior (i.e., geometrical properties, pressure gradient, and heat flux) were considered to characterize the performance of the heat sink under investigation. 

The results were used to develop simple polynomial correlations that enable the evaluation of the most important parameters that characterize the rectangular channels as a function of the aspect ratio. More specifically, any generic parameter *Y* was evaluated as follows:

(12) $$Y=\overset{4}{\underset{j=0}{\sum }}C_{j}\beta^{j}$$

where *C_j_* are the coefficients of the polynomial correlation, which are reported in Table 1 for the most important parameters investigated in the present analysis. 

Particularly, the coefficients presented in Table 1 enable the evaluation of the following parameters: the friction factor-Reynolds number product and the Nusselt number.

The friction factor-Reynolds number product was defined as follows:

(13) $$fRe=-\frac{2D_{h}}{\mu W}\frac{\partial p}{\partial z}$$

being *f* the Darcy Weisbach friction factor. 

The average Nusselt number for the cross-section was evaluated as follows [24]:

(14) $$Nu=\frac{hD_{h}}{\lambda }$$

where *h* was the convective heat transfer coefficient. 

The mean velocity of the fluid flow in each channel was evaluated by using the balance equation:

(15) W= [(fRe μ L2K ρ Dh2)2+2 ΔpK∞ ρ]1/2− fRe μ L2K ρ Dh2

where *K* was evaluated as follows:

(16) $$K=\;\sum_{i}K_{i}\;+K_{\infty }$$

where *K_i_* is the minor losses (i.e., losses due to the inlet and outlet) and *K_∞_* the pressure factor.

The mass flow rate $\dot{m}$ in a single channel was:

(17) $$\;\dot{m}=\rho Wab$$

The increase in the air bulk temperature between the inlet and outlet sections was:

(18) $$\Delta \theta_{\mathrm{bulk}}=\frac{Q}{N\dot{m}c_{p}}$$

where *Q* was the exchanged total power, *N* the number of channels, and *c_p_* the specific heat at a constant pressure.

The limiting parameter that must be checked to ensure the reliability and safety of the heated component is the maximum temperature of the substrate; for instance, in silicon electronic devices, the maximum temperature must not exceed about 120 °C [34]. The maximum temperature depends on many physical and geometric parameters, but if the dimensions of the heat sink and the thermal power are assigned, the maximum temperature depends only on the number of channels built in the heat sink, which in turn determines the aspect ratio.

The maximum temperature of the substrate *θ_s_*, reached in the outlet section of the fluid in the heat sink, was evaluated as follows:

(19) $$\theta_{s}=\theta_{in}+\frac{Q}{N}\left(\frac{1}{\dot{m}c_{p}}+\frac{1}{hLP_{h}}\right)$$

To evaluate the performance of the heat sink, it is crucial to calculate the pumping power, which was defined as [6]:

(20) $$\Pi =M\frac{\Delta p}{\varrho }$$

where $\dot{m_{tot}}$ was the total mass flow rate.

The performance of the heat sinks was also evaluated in terms of thermal resistance *R_th_*, which was defined as follows [6]:

(21) $$R_{th}=\frac{\theta_{s}-\theta_{in}}{Q}=\frac{1}{N}\left(\frac{1}{\dot{m}c_{p}}+\frac{1}{hLP_{h}}\right)$$

The simplified model above described was validated by comparing the results obtained by applying the simplified model with experimental and numerical data available in the literature [4,35]. The comparison was carried out by considering the thermal resistance of the heat sink, which was evaluated as reported in [4]. Because the heat sink investigated in [4,35] was made of silicon, the H1,4 boundary condition was considered for the simplified model.

**Figure 3:**
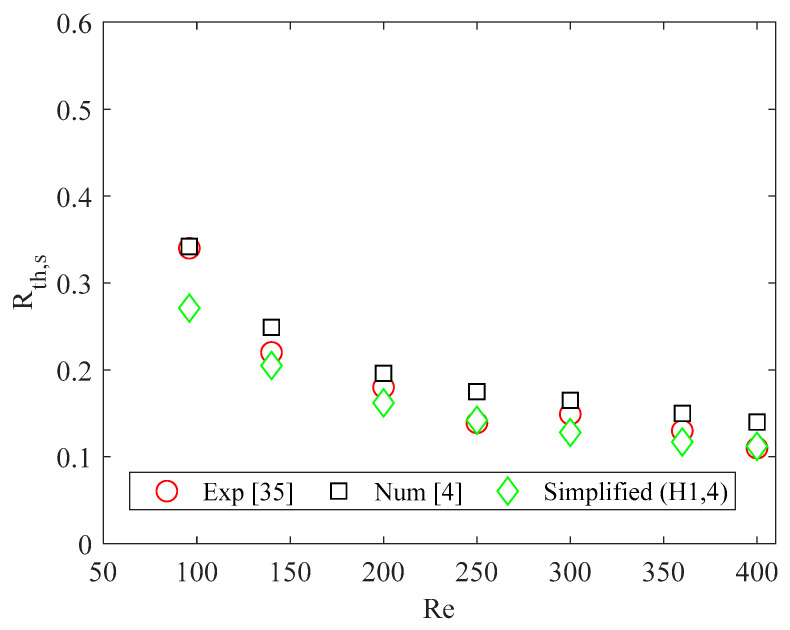
Thermal resistance of heat sink (°C cm^2^/W): comparison between results of the simplified model, numerical data obtained by considering conjugate heat transfer [4], and experimental data [35]. Reproduced with permission from [4].

A good agreement was found for *Re* > 96, as shown in Figure 3. The difference observed for *Re* = 96 can be explained by considering that for this value of the Reynolds number the increase in the fluid bulk temperature was high, therefore the results obtained by applying the simplified model were not accurate.

## 3. Results and Discussion

The simplified model described and validated in the previous section (i.e., Equations (12)–(21)) were used to evaluate the performance of the heat sink under different operating conditions. The analysis was carried out by considering several heat sink sizes, namely, *B* = *L* = 2.5 mm, *B* = *L* = 5 mm, *B* = *L* = 10 mm, and *B* = *L* = 20 mm. The sides of the rectangular channels and the wall thicknesses were varied to make applicable the assumptions presented in the previous section. Specifically, for the heat sink with *B* = *L* = 2.5 mm, the height of channels *b* and the wall thickness were equal to 1 × 10^−4^ mm and 2.4 × 10^−5^ mm, respectively, while the channel width *a* ranged between 2.48 × 10^−3^ mm and 1 × 10^−4^ mm. For the heat sink with *B* = *L* = 5 mm, the height of channels *b* and the wall thickness were equal to 2 × 10^−4^ mm and 5 × 10^−5^ mm, respectively, while the channel width *a* ranged between 4.95 × 10^−3^ mm and 2 × 10^−4^ mm. For the heat sink with *B* = *L* = 10 mm, the height of channels *b* and the wall thickness were equal to 4 × 10^−4^ mm and 1 × 10^−4^ mm, respectively, while the channel width *a* ranged between 9.9 × 10^−3^ mm and 4 × 10^−4^ mm. For the heat sink with *B* = *L* = 20 mm, the height of channels *b* and the wall thickness were equal to 8 × 10^−4^ mm and 2 × 10^−4^ mm, respectively, while the channel width *a* ranged between 1.98 × 10^−2^ mm and 8 × 10^−4^ mm.

For each heat sink size considered in the present study, the number of channels ranged between 1 and 20.

A heat flux *q* = 1500 W/(m^2^ K) was applied to each considered heat sink.

Air was considered as a working fluid; the pressure gradient was assumed to be equal to 400 Pa, while the minor losses were assumed to be equal to 2.5 [36]. The following thermophysical properties were considered: *ρ* = 1.27 kg/m^3^, *c* = 1,005 J/(kg K), *μ* = 1.75 × 10^−5^ Pa s, *λ* = 0.0246 W/(m K), and *Pr* = 0.715.

**Figure 4:**
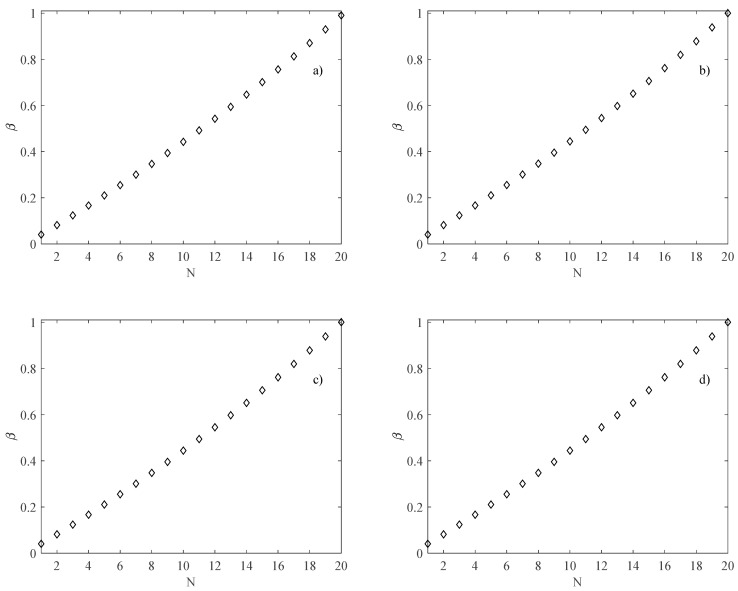
*β* as a function of the number of rectangular channels; (**a**) *B* = *L* = 2.5 mm; (**b**) *B* = *L* = 5 mm; (**c**) *B* = *L* = 10 mm; (**d**) *B* = *L* = 20 mm.

Because the number of channels *N* is related to the aspect ratio *β* (i.e., increasing the number of the channels by keeping the width of the heat sink *B* fixed leads to a variation in the aspect ratio of the channels), the first step of the analysis is to evaluate *β* as a function of *N*. It was observed that the aspect ratio increases with an increasing number of the channels, as shown in Figure 4, where *β* as a function of *N,* is reported for all heat sink sizes. Figure 4 also shows that to analyze only the effects of the channels’ number and the thermal boundary condition, the same trend of *β* was adopted for each heat sink size considered in the present study (i.e., for each heat sink size, both ratios *b*/*L* and *a*/*L* were kept constant).

To adopt the simplified model, the validity of the assumptions made to obtain the correlations developed in the present study must be verified.

**Figure 5:**
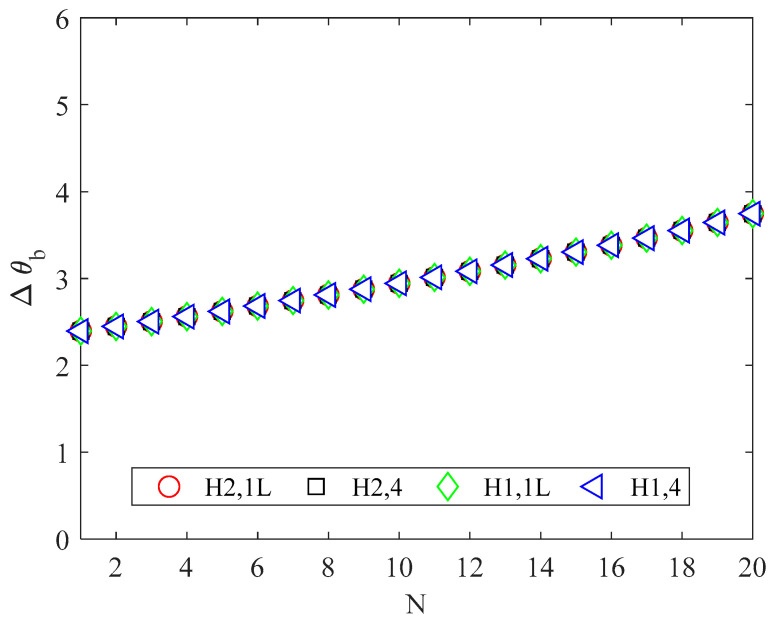
Increase in the air bulk temperature (°C).

To check the validity of the assumption related to the thermophysical properties of the working fluid, the increase in the air bulk temperature must be evaluated. For the parameters considered here, the increase in the bulk temperature of the working fluid was minimal, as shown in Figure 5. Therefore, the simplified model was used for the purpose of the present study.

Figure 5 shows that the increase in the air bulk temperature is higher for a high number of channels; this trend can be explained by considering that the mass flow rate for each channel decreases by increasing the number of channels because of the decrease in the fluid mean velocity and cross-sectional area.

To verify the assumption of a fully developed flow, the lengths of the dynamic and thermal entrances have to be evaluated. An estimation of the dynamic and the thermal entrance lengths was provided by the correlations proposed in the literature [24,30,31,32,33].

After checking the reliability of the mathematical model, the performance of the heat sinks was evaluated.

**Figure 6:**
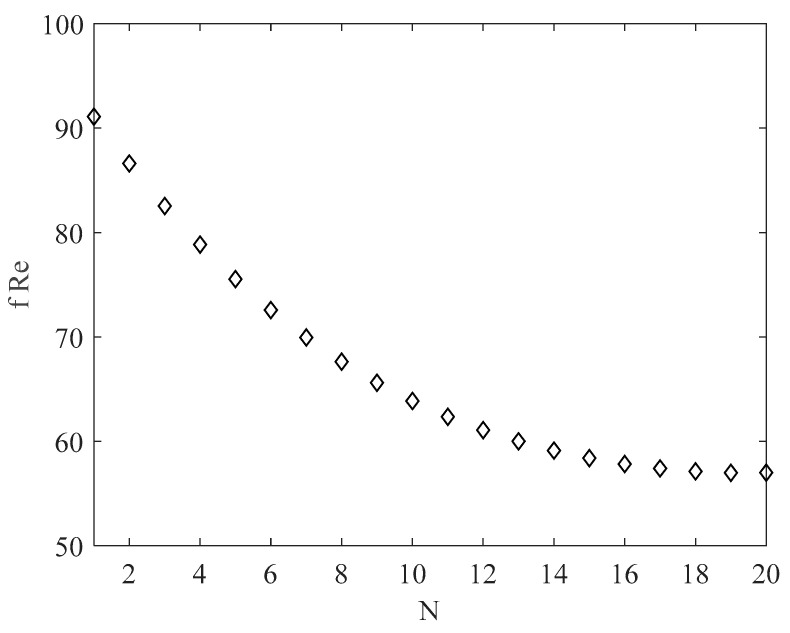
*f Re* as a function of the number of rectangular channels.

The main parameter affecting the velocity and mass flow rate is the friction factor; it was found that the product *f Re* significantly decreased with the number of channels, due to the increase in the friction factor and the decrease in the hydraulic diameter [24]. The *f Re* product as a function of the channel number is presented in Figure 6. Because it was evaluated by means of Equation (12), it depends only on the aspect ratio (i.e., the product *f Re* was the same for each heat sink size considered here.

It must be stated that the increase in the number of channels involves increasing the aspect ratio, which in turn implies a sharp increase in the friction factor and a decrease in the total flow rate.

**Figure 7:**
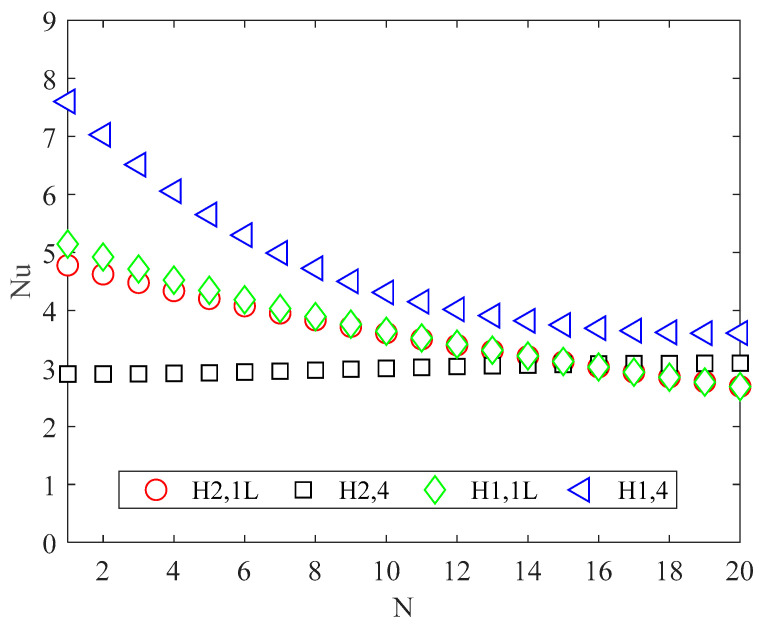
Nusselt number for different channel numbers and conditions.

The Nusselt numbers, evaluated by using Equation (12), are shown in Figure 7 for all the thermal boundary conditions. When only one of the four sides in the cross section is heated, the Nusselt number decreases significantly with an increasing number of channels. When the perimeter is fully heated (version 4), the Nusselt number slightly increases in the H2 boundary condition, while in the H1 boundary condition it strongly decreases as the number of channels increases. 

The differences between the values given by the polynomials (12) and the corresponding values published in the bibliography [28,29] are always less than 0.25%, as shown in Table 2.

**Figure 8:**
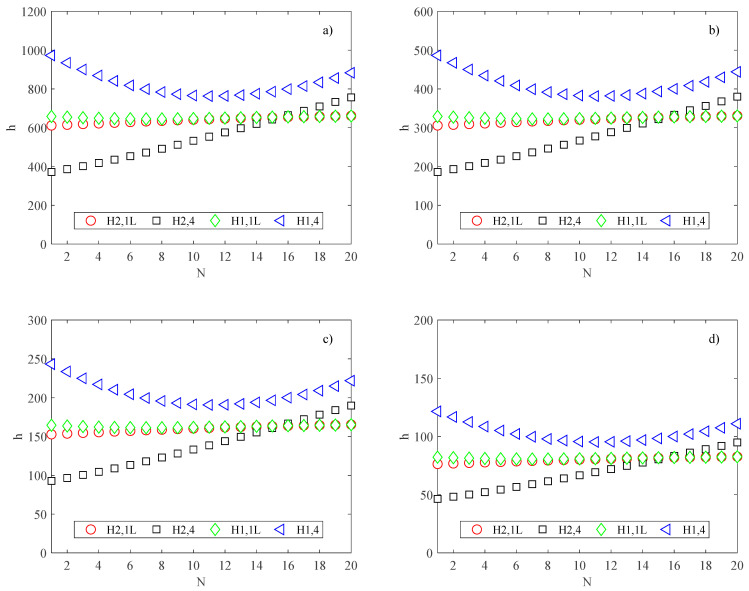
Heat transfer coefficient (W/m^2^K) for different channel numbers and conditions; (**a**) *B* = *L* = 2.5 mm; (**b**) *B* = *L* = 5 mm; (**c**) *B* = *L* = 10 mm; (**d**) *B* = *L* = 20 mm.

Due to the decrease in the hydraulic diameter *D_h_* when increasing the number of channels, the convective heat transfer coefficients present different trends; particularly, for the H2,4 boundary condition, *h* is a monotonic increasing function, while for the H2,1L boundary condition, it slightly increases as the number of channels increases, as shown in Figure 8. 

On the contrary, for the H1,4 boundary condition, the convective heat transfer coefficient decreases with an increase in the number of channels for *N* < 11, while it increases for *N* > 11. For the H1,1L boundary condition, *h* decreases slightly as the number of the channels increases.

**Figure 9:**
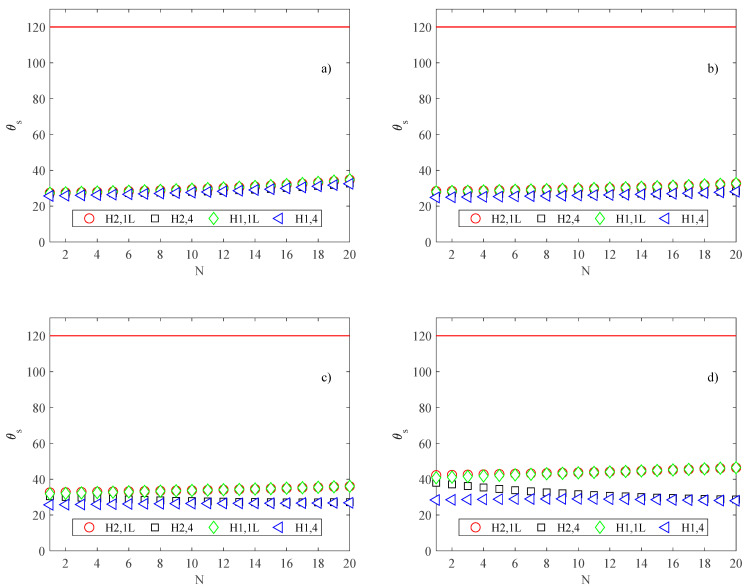
Maximum substrate temperature (°C); (**a**) *B* = *L* = 2.5 mm; (**b**) *B* = *L* = 5 mm; (**c**) *B* = *L* = 10 mm; (**d**) *B* = *L* = 20 mm.

However, in all operating conditions analyzed in the present study, the convective heat transfer coefficient does not increase enough to allow an improvement in the cooling effect; that is, the effect related to the decrease of flow prevails on the increase of the heat transfer coefficient, and the maximum temperature reached by the substrate increases as the number of channels increases, in all four situations of H1 and H2 examined, as shown in Figure 9, where the maximum substrate temperature is presented.

It is important to note that because the convective heat transfer coefficient depends on the hydraulic diameter, it does not depend only on the aspect ratio but also on the channel height *b* (i.e., *D_h_* = 4*A*/*P* = 2*ab*/(*a* + *b*) = 2*b*/(1 + *β*)). Therefore, although the trends of *h* as a function of *N* were the same for each heat sink size considered here, their values were different, as shown in Figure 8.

The substrate temperatures *θ_s_* evaluated by means of Equation (19) as a function of the channel number *N* are presented in Figure 9 for the different heat sink sizes.

As expected, the best cooling effect is obtained for version 4, when the wall temperature is uniformly distributed on all perimeters of the cross-section. The version H1,4 (highly conductive walls) allows for maintaining cooler working conditions (for comparison, Figure 9 also shows the limiting temperature for electronic devices as equal to 120 °C [34]).

By comparing the results for the different heat sink sizes considered in the present study, it can be deduced that for a small heat sink size, the substrate temperature is an increasing function of the channel number, while for a large heat sink, the substrate temperature slightly decreases as the number of channels increases. This trend can be explained by considering that large heat sinks are characterized by low convective heat transfer, as shown in Figure 8.

**Figure 10:**
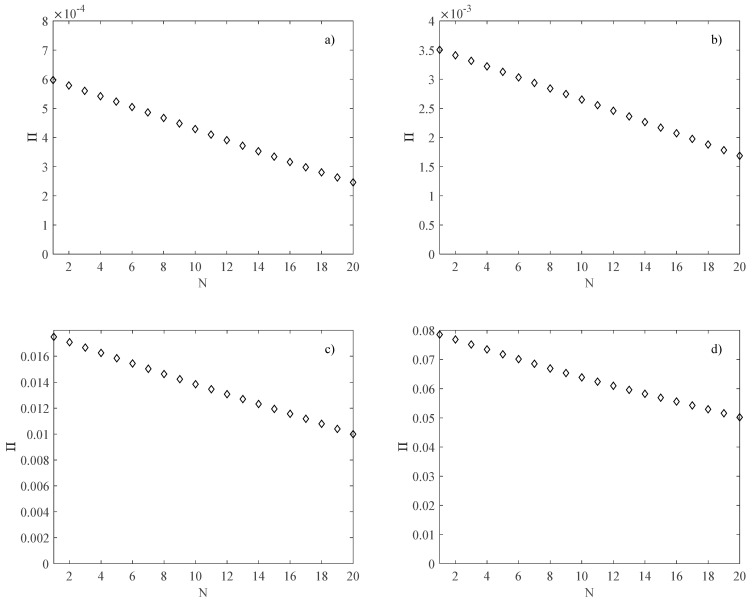
Pumping power (W); (**a**) *B* = *L* = 2.5 mm; (**b**) *B* = *L* = 5 mm; (**c**) *B* = *L* = 10 mm; (**d**) *B* = *L* = 20 mm.

The performance of the heat sink in terms of pumping power was evaluated by means of Equation (20), is depicted in Figure 10 as a function of the channel number *N*. As expected, the pumping power decreases as *N* increases due to the decrease in the total mass flow rate.

**Figure 11:**
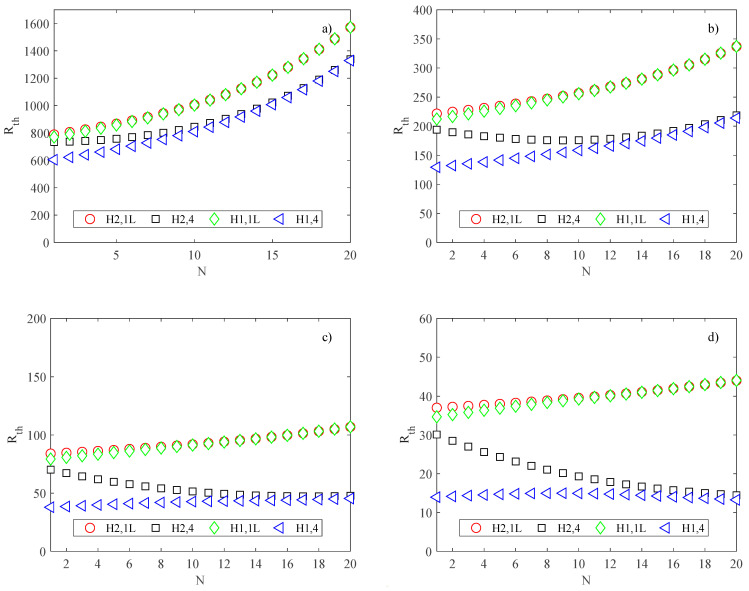
Thermal resistance of the heat sink (K/W); (**a**) *B* = *L* = 2.5 mm; (**b**) *B* = *L* = 5 mm; (**c**) *B* = *L* = 10 mm; (**d**) *B* = *L* = 20 mm.

The cooling performance of the heat sink is well represented also by its thermal resistance, defined in Equation (21), and shown in Figure 11 as a function of the number of channels, for all the thermal boundary conditions considered in the present study.

The boundary condition H1,4 corresponds to the lower values of thermal resistance; the increase in the number of channels is responsible for worse cooling performance.

To evaluate the influence of the geometric properties, different values of channel height, channel width, and wall thickness were considered. Moreover, the influence of heat flux and pressure gradient was investigated. The analysis of the results obtained for these different operating conditions leads to the same conclusion.

## 4. Conclusions

In the present study, a simplified model for the evaluation of the cooling performance of heat sinks with rectangular minichannels under different operating conditions is presented. The thermal boundary conditions and the number of channels were the main parameters considered.

The outcomes clarify that the performance of the heat sink is significantly affected by both the thermal boundary conditions and the number of channels. The main results can be summarized as follows: the product *f Re* decreases significantly as the number of channels increases due to the increase in the aspect ratio; the Nusselt number decreases significantly with an increase in the number of channels when only one side of the rectangular channels is heated (i.e., version 1L) for both the H1 and H2 boundary conditions.

When all sides of the rectangular channels are heated (i.e., version 4), the Nusselt number slightly increases in the H2 boundary condition while it decreases significantly in the H1 boundary condition as the number of channels increases; and the best cooling effect, in terms of the maximum substrate temperature and thermal resistance of the heat sink, is obtained for highly conductive walls (i.e., version H1,4).

The simplified model presented here offers a simple tool for technicians and designers that enables the evaluation of the performance of heat sinks with rectangular channels without resorting to numerical simulation, which requires high computational effort and cannot be generalized to a wide range of channel configurations. Obviously, for a more accurate analysis, the conjugate heat transfer must be studied.

## Figures and Tables

**Table 1:** Coefficients in polynomial Equation (12).

*Y*	*C_0_*	*C_1_*	*C_2_*	*C_3_*	*C_4_*
*f Re*	96	−127.99	167.70	−109.13	30.41
*Nu_H2,1L_*	4.94	−4.13	3.44	−2.293	0.73
*Nu_H2,4_*	2.91	−0.20	1.70	−2.11	0.79
*Nu_H1,1L_*	5.39	−6.25	7.37	−5.59	1.77
*Nu_H1,4_*	8.24	−16.75	24.69	−17.86	5.29

**Table 2:** Comparison between present results (i.e., Equation (12)) and data available in the literature.

	NuH2,1L		NuH2,4		NuH1,1L		NuH1,4	
β	Equation (12)	[29]	Equation (12)	[29]	Equation (12)	[28]	Equation (12)	[28]
0.1	4.559	4.558	2.905	2.907	4.828	4.820	6.790	6.785
0.125	4.473	4.471	2.908	2.909	4.708	4.700	6.493	6.49
0.2	4.234	4.233	2.922	2.922	4.388	4.380	5.738	5.738
0.25	4.090	4.089	2.936	2.935	4.202	4.196	5.332	5.331
1/3	3.870	3.87	2.964	2.964	3.935	3.931	4.799	4.795
0.5	3.494	3.494	3.021	3.022	3.514	3.513	4.132	4.123
2/3	3.181	3.179	3.063	3.064	3.188	3.186	3.797	3.79
0.75	3.042	3.041	3.076	3.077	3.046	3.044	3.704	3.701
5/6	2.914	2.913	3.084	3.085	2.915	2.913	3.643	3.6453
1	2.690	2.686	3.090	3.091	2.688	2.686	3.615	3.608

## Data Availability

No repositories involved or shared.
